# Tumor of Parotid Gland

**Published:** 1887-06

**Authors:** 


### Tumor of Parotid Gland—Entire Gland Removed—Recovery.

John H., Thamesville, Ontario; aged twenty-eight; carpenter. Admitted November 23, 1886.

*History.*—Family history good. Five years ago he noticed a small hard lump below his right ear. Its growth was slow and gave the patient no discomfort until May, 1885, when it became the seat of severe, sharp, darting pain, on account of which he had it removed by Dr. Smith, of Chicago. One month after the operation he noticed a bunch in the cicatrix, which grew very rapidly, attaining nearly its present size in six months. At intervals he has a sharp darting pain in the tumor at night, which is aggravated after exposure.

*Present Condition.*—General health good. The right side of his face and neck presents a large tumor. Its base extends from the zygomatic arch downwards below the angle of the jaw, and encroaches forward on the face, and extends posteriorly to the mastoid process. It involves the lobe of the ear, and has pushed it outward several inches from its normal position. The mass is firm and hard, and has a very marked nodulated appearance. The integument covering it is normal excepting at the site of the former operation, where it is red and dark-colored and smooth and shining. It is slightly movable, and does not seem to involve the jaw, but from its size and position the movements of the lower jaw are seriously interfered with. His hearing on the side of the tumor has become defective. There is no pain or tenderness on pressure, but there is a dull pain mostly at night, and at times severe shooting pains from the tumor into his ear. The glands of the neck are not enlarged. Facial paralysis since former operation.

November 29: Was shown at clinic. The tumor being well defined and not firmly fixed to the base of the skull, the case was a favorable one for operation. When under the anæsthetic, an incision was made down on the tumor from the zygoma to the angle of the jaw, a second cut was made from the first, back over the mastoid process. Flaps of the integument were dissected up, and the tumor dissected out. Another large fibroid growth, distinct from the former, was removed through a second opening made along the posterior border of the sterno-mastoid muscle.

The great depth of the tumor made the operation difficult and hazardous. It extended under the skull, and involved the ligaments of the inferior maxillary articulation, and was firmly bound to the under surface of the temporal bone. These fibrous connections were so dense that the knife had to be used at every step of the operation, the difficulties of which were greatly increased by the profuse hæmorrhage which was constantly occasioned by the division of the numerous blood-vessels. Following the last rapid sweeps of the knife, the hæmorrhage was very great for an instant; the spouting vessels were quickly seized with catchforceps and ligated. The temporal, facial, internal maxillary, and external carotid arteries, and many other smaller but very active vessels were divided.

After all the bleeding points had been secured, and the cavity thoroughly cleansed, the common carotid artery, the internal jugular vein, the hypoglossal, gustatory, pneumogastric, and cervical nerves, the styloid and mastoid processes, were plainly in view in the bottom of the wound, and the transverse processes of the upper cervical vertebræ could be easily felt.

The wounds were thoroughly irrigated with bichloride solution; a drainage tube placed in each; then closed with numerous silk sutures, and dressed with oiled-silk, bichloride gauze, and sheet cotton.

December 2: Wound has healed superficially troughout its greater part. There is some pain when he swallows, but not at any other time.

December 5: Sutures, ligatures, and drainage tubes removed. Cavities rapidly filling up. Discharge free and abundant.

December 9: Cavities of the wounds nearly closed. Discharge slight. Paralysis of face same as before the operation.

December 15: Posterior wound healed. A small depression remains open in the anterior wound, which healthy granulations are filling up.

December 20: Small granulating surface not healed, but it is closing so rapidly that the patient is permitted to leave the hospital.

NOTE.—Highest temperature, November 30, 102.2°. The patient sends information (March 26) that the wound is thoroughly healed, and as yet no appearance of recurrence of tumor. —*Exchange.*

---

COLLODION OF IODOFORM.—This preparation has been successfully used for the relief of neuralgia, and is usually prepared by dissolving one part of iodoform in fifteen parts of collodion. Occasionally ten per cent. or even twenty-five per cent. solutions have been employed.

